# The implementation of a perioperative medicine for older people undergoing surgery service: a qualitative case study

**DOI:** 10.1186/s12913-024-10844-0

**Published:** 2024-03-15

**Authors:** Margot E Lodge, Jugdeep Dhesi, David JH Shipway, Philip Braude, Catherine Meilak, Judith Partridge, Nadine E Andrew, Velandai Srikanth, Darshini R Ayton, Chris Moran

**Affiliations:** 1 National Centre for Healthy Ageing, Melbourne, Australia; 2 Peninsula Clinical School, Central Clinical School, Monash University, Melbourne, Australia; 3 Alfred Health, Melbourne, Australia; 4 Guy’s and St Thomas’ NHS Foundation Trust, London, UK; 5 King’s College London, London, UK; 6 CLARITY (Collaborative Ageing Research) group, North Bristol NHS Trust, Bristol, UK; 7 University of Bristol, Bristol, UK; 8 East Kent Hospitals University NHS Foundation Trust, Canterbury, UK; 9 Peninsula Health, Frankston, Australia; 10 Health and Social Care Unit, Monash University, Melbourne, Australia; 11 School of Public Health and Preventive Medicine, Monash University, Melbourne, Australia

**Keywords:** Perioperative care, Older people, Implementation science, Qualitative research

## Abstract

**Background:**

The international scale and spread of evidence-based perioperative medicine for older people undergoing surgery (POPS) services has not yet been fully realised. Implementation science provides a structured approach to understanding factors that act as barriers and facilitators to the implementation of POPS services. In this study, we aimed to identify factors that influence the implementation of POPS services in the UK.

**Methods:**

A qualitative case study at three UK health services was undertaken. The health services differed across contextual factors (population, workforce, size) and stages of POPS service implementation maturity. Semi-structured interviews with purposively sampled clinicians (perioperative medical, nursing, allied health, and pharmacy) and managers (n = 56) were conducted. Data were inductively coded, then thematically analysed using the Consolidated Framework for Implementation Research (CFIR).

**Results:**

Fourteen factors across all five CFIR domains were relevant to the implementation of POPS services. Key shared facilitators included stakeholders understanding the rationale of the POPS service, with support from their networks, POPS champions, and POPS clinical leads. We found substantial variation and flexibility in the way that health services responded to these shared facilitators and this was relevant to the implementation of POPS services.

**Conclusions:**

Health services planning to implement a POPS service should use health service-specific strategies to respond flexibly to local factors that are acting as barriers or facilitators to implementation. To support implementation of a POPS service, we recommend health services prioritise understanding local networks, identifying POPS champions, and ensuring that stakeholders understand the rationale for the POPS service. Our study also provides a structure for future research to understand the factors associated with ‘unsuccessful’ implementation of a POPS service, which can inform ongoing efforts to implement evidence-based perioperative models of care for older people.

**Supplementary Information:**

The online version contains supplementary material available at 10.1186/s12913-024-10844-0.

## Background

The number of older people requiring surgery is increasing [1, 2]. Delivering perioperative care to older people is complex, with interactions required between multiple people and systems, within and outside the hospital environment [1, 3, 4]. This contributes to variation in the delivery of perioperative care of older people internationally, with variations existing even in geographically approximate hospitals with similar health systems [5, 6].

Person-centred, multidomain, and multidisciplinary comprehensive geriatric assessment and optimisation (CGA)-based models of perioperative care have demonstrated improvements in outcomes [1, 7]. The perioperative medicine for older people undergoing surgery (POPS) service is one such CGA-based perioperative model of care. In the vascular surgery setting, a randomised controlled trial (RCT) of 176 patients reported that care from the POPS service was associated with shorter hospital length of stay, lower incidence of delirium, cardiac, bowel and bladder complications, and less post-discharge institutionalisation than the control model of care [8]. The results of subsequent observational studies suggest that services like POPS may have benefits outside the vascular surgery setting and lead to improvements in length of stay, 1-year postoperative mortality, hospital-acquired geriatric syndromes, rates of postoperative medical complications, and quality-adjusted life-years [9–15]. This work has informed international best practice guidelines that recommend CGA-based perioperative models of care [3, 16, 17]. However, despite this evidence and recommendations, widespread uptake of the POPS service has been inconsistent [5, 6]. For example, only 18% of geriatric medicine departments in Australia and New Zealand provided a proactive CGA-based perioperative model of care despite a unanimous belief in the need for this model of care [5].

One reason for the inconsistent scale and spread of innovations like the POPS service is its complexity and the way its elements interact with surrounding context (the clinical, organisational, physical, economic, cultural, and political environments) [18, 19]. Elements of the POPS service have previously been identified using a logic model approach that describes how the multidisciplinary POPS team works with other key stakeholders to deliver CGA in the pre- and postoperative setting [20]. However, implementing the elements of a complex intervention like a POPS service can be challenging unless there is an understanding of its interacting components, groups and behaviours, and their relationships to surrounding context [18, 19]. Having an in-depth, structured understanding of the factors that influence implementation can aid those planning implementation to develop strategies to optimise facilitators and mitigate barriers to change, and support successful implementation [19, 21].

Recently, there have been calls for implementation science to be applied to improve the perioperative care of older people [1, 22, 23]. Previous work exploring the implementation of models of care like the POPS service has examined single-site implementation or a small scope and number of disciplinary stakeholders [24, 25]. We therefore aimed to describe factors that acted as barriers or facilitators to the implementation of a POPS service in three health services in the United Kingdom (UK) with different levels of POPS service maturity. We studied three POPS services of varying levels of service maturity, where implementation had been both achieved and sustained. This suggested factors relevant to implementation in these three health services were more likely to act as facilitators than barriers.

## Methods

### Study design

We conducted a qualitative case study to examine the implementation of POPS services at three health services in the UK. We specifically conducted a *multiple descriptive case study*, which provides a discovery-led approach to describing an intervention and the real-life context in which it occurs [26]. Qualitative case studies differ from clinical case studies (which follow an individual patient of interest) by focusing on an intervention (such as the POPS service) as the phenomenon of interest. In a qualitative case study, individual ‘cases’ are used to explore the phenomenon of interest [26]. We defined the three ‘cases’ of our qualitative case study as each of the three health services with established POPS services.

This methodology allowed us to conduct an in-depth exploration of the implementation of POPS services, with each health service acting as a case through which we could explore implementation. Including multiple cases with different levels of implementation maturity improves the transferability of our findings [26, 27].

We used the consolidated criteria for reporting qualitative research (COREQ) as a reporting guide (see Additional file 1) [28].

### Theoretical framework

Our interview schedule was informed by the POPS logic model [20]. We used the Consolidated Framework for Implementation Research (CFIR) to organise themes identified in our data [29]. The CFIR aims to assist the understanding of factors relevant to implementation – or, “what works where and why” [29]. It has been used for other perioperative interventions including music therapy [30, 31], regional anaesthesia [32], nutrition care [33], clinical handover between the operating room and intensive care unit [34], and perioperative risk assessment and shared decision-making [35]. The CFIR consists of five domains and multiple constructs related to each domain. Not all constructs, nor all domains, are expected to be identified when exploring factors related to implementation [29]. When tailored to the POPS service the domains include factors related to:

- ‘Characteristics’: the nature of POPS itself, e.g., complexity and adaptability;
- ‘Outer setting’: context external to the health service, e.g., government strategies;
- ‘Inner setting’: context within the health service, e.g., health service culture;
- ‘Characteristics of individuals’: people implementing and delivering the POPS service, e.g., POPS geriatricians;
- ‘Processes’: how POPS is implemented, e.g., planning and executing implementation.

### Case selection, sampling and recruitment

We identified three cases that differed in size, geographical location, operational approach, and implementation maturity of POPS services to ensure a deep understanding of implementation factors and enhance transferability of our findings. Author JD, who pioneered the POPS service and is the clinical lead at case 1, used her knowledge of POPS services in the UK to inform selection of the cases to ensure diversity in case characteristics.

Within each case, we used purposive and snowball sampling methods to select participants. First, we purposively identified a list of 16 core clinical and managerial disciplines we wished to interview. This list was developed to include a range of stakeholders with different perspectives on the implementation of POPS services (see Additional file 2). We provided this list to the clinical leads at the cases (JD, JP, DS, PB, CMe). The clinical leads provided the name and contact details of individuals in each of these roles and supported author ML to contact each person via email. We utilised snowball sampling processes by asking participants to consider if there were other stakeholders not identified through the original sampling process, who may have relevant insights. These individuals were then invited to participate.

ML, the interviewer, did not have an established relationship with participants. She corresponded with potential participants via email to recruit and organise interviews. Potential participants were informed of ML’s background as a geriatrician and PhD candidate via introductory email. Participants were offered the opportunity to ask questions of ML before consent was recorded at the start of the interview.

### Setting

The cases in our study were three health services in NHS (National Health Service) England, a publicly-funded healthcare system that provides the majority of healthcare in England, delivering most services free to users at the point of access [36]. Inpatient care is provided in hospitals that are grouped and administered as ‘Trusts’.

#### Case 1

Case 1, in central London, is a tertiary-level care provider that includes five hospitals. The POPS service is delivered at two hospitals within the health service, with a combined bed base of approximately 1,200 beds. The POPS service provides input for vascular, orthopaedics, gynaecological oncology, thoracic, urological, and colorectal patients. Care is delivered to inpatients and outpatients contemplating surgery. The health service pioneered the development of the POPS service in 2003.

#### Case 2

Case 2 is a tertiary-level care provider that includes one main hospital with approximately 1,000 beds and is the region’s major trauma unit. This health service’s POPS (referred to locally as ‘G-POC’ – Geriatric Perioperative Care) service provides input for vascular, colorectal, urological, breast oncoplastic, major trauma, burns, and orthopaedic patients. The POPS service is mostly delivered in the inpatient setting, with some input in outpatient preoperative assessment clinics. The POPS service was implemented in 2017. Case 2’s POPS clinical leads spent time training at case 1 as registrars under the supervision of the case 1 clinical leads.

#### Case 3

Case 3 includes three hospitals. The POPS service is provided at one of these hospitals with approximately 300 beds and a General Practitioner-led urgent treatment centre but no Emergency Department. The POPS service supports urological and vascular surgical inpatients and provides care in the outpatient preoperative assessment clinic. The POPS service was established here in 2019. Case 3’sPOPS clinical lead was previously involved with the translation of the POPS service to a district general hospital with the support of the case 1 POPS clinical leads and was subsequently recruited to establish a new POPS service at case 3.

### Data collection

We designed a semi-structured interview schedule (see Additional file 3), using the previously published POPS logic model [20] as the underlying theory. The published POPS logic model consists of seven categories (inputs, core components, mechanisms (process changes), contextual factors, short-term outcomes, long-term outcomes and wider impact of the POPS service), and our initial interview schedule was constructed using these categories. We developed specific questions to explore each of the categories, utilising the body of the published logic model to guide the questions. Probing questions were used to explore participants’ responses. The interview guide was reflexively revised following interviews based on discussions between ML, DA, and CM (see Additional file 4). Revisions included shifting from focusing on specific categories identified in the published POPS logic model to exploring participants’ broader experience of POPS services. This resulted in less-specific questions and enabled a more flexible approach to considering what participants believed about barriers and facilitators to implementation. Opening questions were standardised across all interviews and further prompts were utilised as required.

Interviews were scheduled for 30 min. Participants could choose to shorten or lengthen this time based on their availability and willingness to explore topics more deeply. Participants were interviewed once. Interviews occurred between April and November 2021, with participants from case 1 interviewed first, then case 2 and case 3. ML conducted all the interviews with DA observing three initial interviews to assist in note taking, debriefing and revising the interview guide.

Interviews were audio and video recorded using the Zoom videoconferencing platform. Audio from the interviews was transcribed and imported into NVivo software (QSR International Pty Ltd., release 1.4.1 March 2021). The interviewer (ML) completed field notes during the interview and maintained a reflexive diary following each interview. Transcripts were not returned to participants.

### Data analysis

Multiple rounds of coding were utilised. First, ML and CM independently conducted open coding of 20 transcripts using NVivo. The purpose of this stage of the analysis was to classify discrete pieces of data into initial categories. ML and CM then used an axial coding process to find connections and relationships between the open codes. ML and CM met through this stage of the analysis process to compare and explore their codes and ensure coding consistency. ML then coded the remainder of the transcripts, using open and axial coding processes, with the option to add additional codes as required. Researchers ML, CM, and DA undertook peer debriefing and reviewed the coding process frequently, meeting to discuss themes.

ML then deductively mapped our codes to the CFIR constructs within each case. This informed the development of themes and subthemes for each case. These were compared across cases and final cross-case themes identified by ML, CM, and DA. These themes were then mapped back to the CFIR to ensure coherence and consistency with the conceptual domains of the CFIR. Clinical leads provided written feedback on the interpretation of how themes mapped to the CFIR. Other study participants were not involved in checking results.

### Ethical approval

Ethical approval for this study was obtained from the Health Research Authority (HRA) and Health and Care Research Wales (HCRW) (REC reference: 21/HRA/0362). The study was also registered with Monash University Human Research Ethics Committee (Project Number: 36,404). All methods were carried out in accordance with relevant guidelines and regulations.

### Research team and reflexivity

Our research team consists of clinical, qualitative methods, and implementation science experts with details of our roles and experience in Additional file 5. We addressed reflexivity throughout the research process by utilising field notes, written reflections, and collaborative working within our multidisciplinary team of researchers which facilitated questioning of assumptions and decision-making processes [37, 38]. The lead investigator (ML) is a geriatrician without a working relationship to participants of the three cases, and who has a belief in the benefits of CGA-based perioperative models of care. Other members of the team (DA) are non-clinicians and agnostic about the benefits. Some members of our research team were involved in identifying potential study participants with whom they had working relationships. These team members were not present for the interviews and did not have access to interview transcripts.

## Results

### Participant characteristics

Across the three cases, 131 people were invited to participate with 56 people (43%) agreeing. Not all of the 16 disciplines that we aimed to interview were present in each case. Of the potential disciplines, 16/16 (100%) participated from case 1, 7/9 (78%) participated from case 2 and 12/13 (92%) participated from case 3. The response rates and discipline of participants can be found in Additional file 2. Interviews ranged from 18 to 83 min in length.

### Factors relevant to implementation

We present our themes as mapped to the CFIR. Each domain has a table outlining the identified constructs, POPS-specific definitions, and details of themes, supported by illustrative quotations. Additional quotations can be found in Additional file 6.

#### Domain 1: intervention characteristics

Themes mapped to two constructs: evidence strength and quality, and adaptability (Table 1, Additional file 7).

**Table 1:** Intervention characteristics: constructs with definitions, themes, and illustrative quotations

Construct	POPS-specific definition	Theme	Illustrative quotation
Evidence strength and quality	Clinician and manager perceptions of the evidence supporting the POPS service. Includes ‘external sources’ of evidence (e.g., peer-reviewed literature) and ‘internal sources’ (e.g., local patient outcomes data from the participants’ health services).	Belief in the evidence for the POPS service supports implementation, with external evidence especially important at sites wanting to newly implement POPS services.	“I had seen the data, I’d seen the research and I’d seen it in practice … it made sense to me that you needed to take a multidisciplinary approach to yield an acceptable outcome for these patients.” (Case 3, Executive)
Adaptability	The degree that the POPS service can be tailored in order to meet each case’s local needs and capabilities.	The POPS service can be adapted to enable its implementation to be tailored to local needs and resources.	“I don’t believe it’s efficient to make one size fit all for every clinical scenario. I don’t think the intervention needs to be the same.” (Case 2, POPS clinical lead)

POPS, perioperative medicine for older people undergoing surgery

Regarding **evidence strength and quality**, participants in cases 2 and 3 reported that knowledge of external evidence of beneficial patient outcomes (such as that reported by POPS clinicians from case 1) supported early adoption of POPS services. Once the POPS service was implemented, participants from all cases reported spread of the service to additional surgical units was supported by internal evidence of improved outcomes at their health service. The credibility of the POPS clinical leads as developers and communicators of quality evidence was highlighted as an important facilitator of implementation in all cases.

Participants across all three cases reported that **adaptability** of the POPS service was important for implementation. CGA is a core component of the POPS service and participants in all cases described fidelity to a CGA-based model of care, with locally-relevant adaptations to peripheral components. These adaptations related to referral pathways, responsibility for clinical decision-making, and disciplines that comprised the POPS team. In all cases, participants described adaptations made organically during initial implementation and then as POPS spread throughout each health service. Adaptations occurred in response to local needs and capabilities and were enacted by POPS team members, and managerial and executive staff.

#### Domain 2: outer setting

Themes mapped to three constructs: patient needs and resources, external networks, and external policies and incentives (Table 2, Additional file 7).

**Table 2:** Outer setting: constructs with definitions, themes, and illustrative quotations

Construct	POPS-specific definition	Theme	Illustrative quotation
Patient needs and resources	The level of understanding within the health service of the needs of older people undergoing surgery, and the barriers and facilitators to meeting those needs.	Understanding and prioritising the needs and priorities of older people undergoing surgery supports the implementation of POPS services.	“Older people need more holistic care and not just, ‘oh we’ll fix the fracture and send them on their way’ … it is quite hard to look after older people and if we all work together we do a better job … [POPS results in] this team mentality and everyone is enthusiastic.” (Case 2, POPS fellow)
Cosmopolitanism (external networking)	Networking of the health services with other organisations.	At health services without a POPS service, networking with external well-established POPS services and their clinical leads facilitates implementation.	“I don’t think I had a lot of formal training but I’ve learnt as I’ve gone along with the support from [case 1 POPS clinical lead] who has done it before.” (Case 3, POPS clinical lead)
External policies and incentives	The role of external initiatives to spread interventions.	Financial incentives can assist with the implementation of the POPS service but are not an essential factor.	“[To receive the Best Practice Tariff, patients are] supposed to have a frailty score within 72 hours [of admission] so we tagged on that they should get a full geriatric assessment.” (Case 2, physiotherapist)

POPS, perioperative medicine for older people undergoing surgery

Participants across all three cases reported that knowledge of **patient needs and resources**, particularly the unique and complex perioperative needs of older people, facilitated implementation. Participants from all disciplines believed the POPS service met the perioperative needs of older adults.

We found the importance of **external networks** was particularly evident in the networking of cases 2 and 3 with case 1. POPS clinical leads in cases 2 and 3 established connections to POPS clinical leads in case 1. These links enabled their cases to benefit from the professional knowledge and experiences of case 1 POPS clinicians from multiple disciplines. For example, the case 3 occupational therapist received service development support from the case 1 occupational therapist due to links between these cases’ POPS clinical leads.

**External policies and incentives** were relevant for expansion of the POPS service in case 2, beyond its initial surgical units. Here, the major trauma *Best Practice Tariff* (a national financial incentivisation scheme for achieving certain evidence- or guideline-based targets in a specific clinical setting) provided a financial incentive and prompt to redesign care for older people living with frailty, admitted under case 2’s major trauma unit. Cases 1 and 3 are not major trauma units and therefore were unable to access this funding stream.

#### Domain 3: inner setting

Themes mapped to three constructs: networks and communications, implementation climate, and readiness for implementation (Table 3, Additional file 7).

**Table 3:** Inner setting: constructs with definitions, themes, and illustrative quotations

Construct	POPS-specific definition	Theme	Illustrative quotation
Networks and communications	The nature and quality of relationships and connections between individuals, clinical units and teams that interact with the POPS service.	Networks at multiple organisational levels facilitate sharing of vision and define team members’ roles, which enables delivery of POPS services.	“[The POPS clinical lead and I] communicate very effectively and I know historically [at other health services] there’s been fragmentation between the way anaesthetists do their preoperative assessment and the way the POPS team do and we were quite careful to work together to avoid that becoming a problem and have achieved that completely.” (Case 3, anaesthetist)
Implementation climate			
Tension for change	The extent to which clinicians and managers feel the care provided to older people undergoing surgery needs to change.	The presence of an unmet clinical need results in a tension for change that facilitates the implementation of POPS services.	“I think the model of care is quite well suited to our hospital … we don’t have a [physician-led] medical service yet we’re an acute hospital… [with] acute vascular, renal [services].” (Case 3, physiotherapist)
Compatibility	The level of alignment between the POPS service and a clinician or manager’s goals, skill mix and values. The perceived risks and benefits of introducing a POPS service.	POPS services may be perceived as a threat when clinicians do not see a clinical need the service can meet; POPS services are thus viewed as a risk to autonomy or territory.	“The other thing that has been problematic is the relationship between the anaesthetic perioperative team and [POPS]. Some of the anaesthetists think, ‘what’s the point [of POPS assessment]?’ … I think that that can be a barrier to spreading [POPS] because people can use some of this as empire building.” (Case 2, surgeon)
Learning climate	A climate with time and space for leaders to feel and express fallibility, team members to feel valued and able to assist leaders, and which safely enables trial and error.	POPS clinical leads role-model and drive a learning climate that supports implementation.	“[POPS] feels much more supportive than other ward rounds, [it] is much more integrational [sic]. People feel that they’re able to speak up and ask questions so I think that’s what it brings to [improved safety and quality].” (Case 1, surgical matron)
Readiness for implementation			
Available resources	Financial, education, physical and time-based resources that are dedicated for implementation and use of the POPS service.	The implementation of POPS services can be enabled by adequate resources for staffing, financial support and education.	“[Our POPS service is] fortunate … teaching hospitals with good reputations attract good people and so they’re well resourced.” (Case 1, board member)
Access to knowledge and information	The ability for users of the POPS service to be easily educated about what the POPS service is and how to engage with it.	Knowledge and information about the why, what and how of POPS services is necessary for implementation and can be provided through multiple channels according to local needs and capabilities.	“Education’s important. I went to a meeting and [the POPS clinical lead] was talking [about POPS] and it was amazing. I’d never heard anything about it before … That’s very important, when you’re just starting out, to make yourself known widely.” (Case 1, anaesthetist)

POPS, perioperative medicine for older people undergoing surgery

The importance of **networks and communications** was frequently reported by participants in all three cases. Networks existed within the POPS team, as well as between the POPS team and the broader perioperative multidisciplinary team.

Networks and relationships within the POPS team were supported by a flat hierarchy and minimal staff turnover. Participants described flexibility in roles and responsibilities within the team. For example, in cases 1 and 3, POPS doctors and nurses shared responsibility for assessing patients and developing optimisation plans. This facilitated networks with other members of the perioperative multidisciplinary team, because any member of the POPS team could be approached for any issue, regardless of their disciplinary background. This meant referrers to POPS could be patient-centric and not need to make discipline-specific referrals to the POPS team, which increased the ease and comfort of referrers accessing the service.

Conversely, participants in all cases felt that clearly defined roles and responsibilities for those external to the POPS team were important for implementation. This included an understanding of who had responsibility for clinical decision-making and coordinating discharge. The process of identifying and refining these expectations helped build networks, improve accountability, and strengthen communication.

High-quality formal and informal communication was also important, enabling the development of networks and relationships. Communication within the perioperative multidisciplinary team pertained to day-to-day delivery of the POPS service and provided a higher-level understanding of what the service entailed. This developed a shared vision of the POPS service and supported its spread through the organisation.

Across all cases, participants reported implementation was facilitated by the recognition of an unmet clinical need that created a **tension for change**. In all three cases, participants described an unmet need in physician-led inpatient care for older people undergoing surgery. There were some case-specific differences in the nature of the unmet need. In cases 1 and 3, hospitals where POPS services were delivered did not have Emergency Departments. Participants reported this meant fewer general medicine services were available to surgical inpatients at these two hospitals. In cases 1 and 2, participants described an unmet need in outpatient preoperative assessment. Here, they felt a comprehensive review of older people contemplating surgery that focused on optimisation and the provision of shared decision-making, as well as assessment, was lacking.

Participants in all three cases reported that a perceived lack of alignment or **compatibility** between clinicians’ values and the POPS service could be a potential barrier to implementation. Participants in cases 1 and 2 recalled initial concerns the POPS service would impinge upon anaesthetists’ ‘territory’ in anaesthesia-led preoperative assessment clinics. A similar concern was reported by surgeons in case 3 where the executive-led, ‘top-down’ approach to implementing the POPS service was felt to potentially threaten the decision-making autonomy of surgeons.

Despite the perceived risk of incompatibility, participants in cases 1 and 2 reported it did not act as a barrier to implementation. This may be because the POPS service was first implemented in areas of unmet clinical need and therefore did not ‘threaten’ another clinician’s area of active practice. In case 1, this area was the preoperative assessment clinic and in case 2, the acute inpatient ward. By first implementing the POPS service in clinical settings where there was a clear need, the service was quickly embedded in routine care and thus accepted as being compatible to the majority of stakeholders. In case 3, the perception of a lack of compatibility resulted in early and proactive engagement of stakeholders by senior decision-makers. Participants in case 3 reported this facilitated implementation through identification of shared goals, clarification of the POPS service’s role and understanding of the POPS clinical lead’s skills and attitudes.

The **learning climate** across the cases, particularly as role-modelled and driven by the POPS clinical leads, supported the sustainability component of implementation. Participants described flat hierarchies, care and support for colleagues, and strong formal and informal interdisciplinary education. This created an environment of safe and supported learning.

Participants in all three cases highlighted the contribution of adequate **available resources** for implementation. Resources included staff skills, numbers, and work hours. Participants in cases 1 and 3 emphasised that implementation was supported by POPS clinical leads working as clinical champions beyond paid hours. Participants in all cases highlighted the importance of early development of business cases to financially support necessary resources.

Participants in all cases reported **access to knowledge and information** about the POPS service facilitated implementation. However, the focus of information provision varied between cases. In cases 1 and 2, participants highlighted knowledge of local clinical need and information about the way POPS services may meet this. In case 3, participants emphasised the need for information to guide the incorporation of the POPS service into routine care.

Knowledge and information were provided formally and informally. Formal approaches, including educational presentations and provision of written material, were led by geriatricians in cases 1 and 2, and the Chief Executive Officer and Clinical Director of Surgery and Anaesthetics in case 3. Participants described informal knowledge transfer via interpersonal communication between surgical consultants.

#### Domain 4: characteristics of individuals

A theme mapped to one construct: knowledge and beliefs about the intervention. This construct was reported by participants in cases 2 and 3 (Table 4, Additional file 7).

**Table 4:** Characteristics of individuals: construct with definition, theme, and illustrative quotation

Construct	POPS-specific definition	Theme	Illustrative quotation
Knowledge and beliefs about the intervention	Individual clinicians’ understanding of the rationale for the POPS service. The skills and enthusiasm that individual clinicians have in referring to and engaging with the POPS service.	Understanding the rationale for the POPS service drives initial adoption, and positive experience with the POPS service supports enthusiasm for ongoing implementation.	“[One surgical department] recognised they had a terrible length of stay and so contacted [the POPS clinical lead] and said we’ve heard about your service from other departments, would you come and do some sessions for us.” (Case 2, POPS fellow)

POPS, perioperative medicine for older people undergoing surgery

Participants in cases 2 and 3 highlighted that implementation was supported if adopters of POPS services recognised the need to improve perioperative outcomes for older people and understood the POPS service could meet this need. These **knowledge and beliefs about the intervention** were based upon experiences clinicians had caring for older people with specific perioperative needs. Non-clinician participants described similar knowledge, established by personal experience and data. When stakeholders understood POPS services could meet these perioperative needs, they recognised the rationale for the service and this supported implementation.

Implementation was also supported by beliefs in the POPS service generated by participants observing the skills and enthusiasm of POPS clinical leads. This initiated a positive response from clinicians and managers, strengthening belief in POPS services and increasing interactions with the POPS team. Multiple participants described positive experiences shared amongst surgical consultants from different units, further increasing knowledge and beliefs in POPS and enabling spread throughout the health service.

#### Domain 5: process

Themes mapped to two constructs: engaging and champions (Table 5, Additional file 7).

**Table 5:** Process: constructs with definitions, themes, and illustrative quotations

Construct	POPS-specific definition	Theme	Illustrative quotation
Engaging	The process of attracting and involving individual clinicians and managers to implement and use the POPS service.	Engagement of individuals to implement and utilise POPS services is supported by a flexible and proactive approach to involving early adopters from a range of disciplines who see the benefits of the service.	“Several of my vascular [anaesthesia] colleagues … did POPS clinics and worked with [the now POPS clinical lead] when they were all trainees … training together builds that link from the start and you know who these people are and there’s a … relationship already in place.” (Case 1, anaesthetist)
Champions	Individuals who believe in the POPS service, actively associate themselves with the POPS service and are dedicated to what is required to implement the POPS service.	POPS clinical leads are essential champions of the implementation of the POPS service	“[The] two consultants that run the service are quite young, ambitious, quite forward-thinking and I think that really helps, they’re quite ambitious to get geriatrics embedded in trauma and … into the [non-trauma] wards as well. I think their drive, having those very proactive consultants has helped.” (Case 2, physiotherapist)

POPS, perioperative medicine for older people undergoing surgery

In all three cases, key staff stakeholders supported implementation by **engaging** as implementation leaders, with some case-specific differences in the way engagement and implementation occurred. In cases 1 and 2, a ‘bottom-up’ approach to engagement was led by ward-based geriatricians. Geriatricians engaged other clinicians to implement and utilise the POPS service by role-modelling positive skills and attitudes. In case 3, a ‘top-down’ approach occurred, led by the Chief Executive Officer and Clinical Director of Surgery and Anaesthetics, with anaesthetic and surgical consultants also engaging as implementation leaders. Participants in all three cases described early engagement from clinicians from a range of disciplines. These early adopters could see clinical benefits of the POPS intervention demonstrated by implementation leaders, and were keen to utilise the service.

Participants from all three cases identified **champions** who facilitated implementation, with POPS clinical leads the key champions. The clinical leads role-modelled – practically and philosophically – their beliefs in the service. They demonstrated outstanding skills, dedication, and commitment to the service and older people undergoing surgery. Other clinical and managerial personnel also championed implementation, especially in case 3 where ‘top-down’ drivers of implementation were active. Across the cases, these staff included General Managers, Clinical Directors and Executive staff who were enthusiastic about the POPS service, supporting preparation and progression of business cases and influencing senior health service stakeholders (e.g., the Trust Board) to facilitate implementation.

## Discussion

In this study of 56 stakeholders from three contextually different health services, we describe 14 factors that act as barriers and facilitators to the implementation of POPS services. While the 14 factors were common across the cases, the way each factor was addressed varied between cases. Organisations considering implementing a POPS service should prioritise stakeholder engagement by highlighting the rationale for the service. Networks, POPS champions and POPS clinical leads can all assist in this. Clinicians and managers contemplating implementation can take a flexible approach to implementation, driven by local context.

A consistent finding was the importance of clinical and managerial stakeholders engaging with the rationale for POPS services. This finding is congruent with existing theories of implementation and diffusion of innovations that highlight the role of motivating stakeholders, attributing meaning, and achieving consensus [39, 40]. While this finding was common across the cases, the way each case achieved an understanding of the rationale for POPS services varied. For example, some cases used published external evidence of benefit to drive their case, while others used knowledge of local practice to highlight local need. This highlights the value of flexible approaches to achieving stakeholder engagement that respond to local needs and capabilities.

Stakeholder engagement in the rationale for POPS was facilitated by networks and POPS champions. Although networks are well-known to be important for the implementation of innovations [39, 41], less is known about their specific role in implementing POPS services. We observed variation and flexibility in the way cases utilised their networks. This related to both the structure and membership of the networks, which existed at interpersonal, unit, service, and inter-hospital levels. It also related to different network hierarchies that existed across and within cases, including vertical, horizontal, strong, and weak hierarchies [29]. Networks were understood, explored, and enhanced by POPS champions and POPS clinical leads in all three cases. We recommend evaluating existing networks, identifying champions, and using internal and external relationships to develop new networks as required.

The factors we highlight are not an exhaustive list of all those that may need to be considered when implementing POPS services. Similarly, we are unable to be prescriptive about the way specific health services should act upon our results to plan implementation. Although some tools exist to support people to take our results and develop an implementation strategy (e.g., the CFIR-ERIC (Expert Recommendations for Implementing Change) tool), there is a lack of expert consensus and experience with these approaches [42–44]. Furthermore, while our results demonstrate factors relevant to implementation that were shared across our three contextually different cases, the way that each case responded to the shared barriers and facilitators varied. This suggests that a prescriptive, ‘one size fits all’ approach to implementation planning is unlikely to be appropriate for the implementation of a POPS service. As such, we encourage those considering implementing a POPS service to consider whether the factors relevant to implementation that we have identified are similarly relevant to their context, and to then develop a flexible implementation plan that is tailored to their local setting. Future research examining the implementation of POPS services in additional contexts may facilitate the development of POPS-specific implementation strategies.

Our study has several strengths. Our multiple descriptive case study approach and purposive sampling allowed us to examine implementation of POPS services across more than one case, and from the perspective of many disciplines involved in POPS services, increasing transferability of our results. This builds on findings of previous work that has tended to use a narrower approach to the number of health services, participants, or disciplines represented [24, 25]. To enhance qualitative rigour and increase credibility and transferability of results we collected data from a diverse range of stakeholders, asked interview participants to participate in ‘member checking’, and reviewed our results with peer debriefing. We ensured our findings were dependable and confirmable by maintaining an audit trail and using a reflexive diary.

Our study also has some limitations. We chose three cases that had existing POPS services but no cases where POPS services had been established but not sustained. It is therefore possible this bias means there are factors not identified in our study that are associated with unsuccessful implementation. Our results are predominantly facilitators of implementation, likely reflecting our sampling of sustained POPS services. Although we did not aim to evaluate or define ‘effective’ implementation in each of these cases, this may also be important to consider. Future research should include sites where POPS services were unable to be established, to better understand insurmountable barriers to implementation. Linked to this limitation is our ability to only report perspectives of those who responded to our invitations. For example, despite our best efforts, we were unable to recruit any anaesthetists in case 2. The reasons for this are unclear. Our recruitment processes utilised the POPS clinical leads, who held dual roles as colleagues of potential participants and members of our research team. This may have also contributed to non-response bias. Another limitation relates to the spread of POPS services across the cases we studied. Clinical leads at all three cases had existing professional relationships. Given the emphasis participants placed upon networks, it is unclear if these existing professional relationships are essential for implementation. This is an important consideration for those contemplating implementation who do not have professional connections with case 1. We therefore suggest health services considering implementing a POPS service consider the relevance of our findings in the context of their own connections to existing POPS services. Finally, our study was conducted in the context of England’s NHS which may limit the generalisability of some of our findings to healthcare systems that differ significantly from this universal healthcare setting.

Near the end of our study, the CFIR framework we used in our analysis was updated. The revised CFIR is planned to support “longitudinal consistency” with the original CFIR so our results will maintain their relevance and can be mapped to the revised CFIR in the future if desired [45].

## Conclusions

Our qualitative case study of three contextually different health services demonstrated that factors relevant to the implementation of POPS services across include a shared understanding of the rationale for the service, the presence of supporting networks, and inspiring POPS champions and clinical leads. Each health service varied in how they addressed implementation factors, which demonstrates the need for adaptable and context-specific approaches to implementation of a POPS service. We suggest health services contemplating the implementation of POPS services use the findings of our study to inform how they plan and approach implementation of a service such as POPS. We recommend they review our summary list of relevant implementation factors and adopt a flexible approach that recognises the local needs and capabilities of organisations.

### Electronic supplementary material

Below is the link to the electronic supplementary material.

Supplementary Material 1

## Data Availability

The raw qualitative data used for this study are not available to other researchers in accordance with the ethical approvals granted and consent processes followed. The qualitative codes generated from this data are available from the corresponding author on reasonable request.

## Abbreviations

CGA: comprehensive geriatric assessment and optimisation
POPS: perioperative medicine for older people undergoing surgery
RCT: randomised controlled trial
UK: United Kingdom
COREQ: consolidated criteria for reporting qualitative research
CFIR: Consolidated Framework for Implementation Research
NHS: National Health Service
G-POC: Geriatric Perioperative Care
HRA: Health Research Authority
HCRW: Health and Care Research Wales
ERIC: Expert Recommendations for Implementing Change

## References

[1] Partridge JS, Moonesinghe SR, Lees N, Dhesi JK (2022). Perioperative care for older people. Age Ageing.

[2] Fowler A, Abbott T, Prowle J, Pearse R (2019). Age of patients undergoing surgery. Br J Surg.

[3] Centre for Perioperative Care. Guideline for Perioperative Care for People Living with Frailty Undergoing Elective and Emergency Surgery. 2021. https://www.cpoc.org.uk/sites/cpoc/files/documents/2021-09/CPOC-BGS-Frailty-Guideline-2021.pdf. Accessed 31 October 2022. 10.1093/ageing/afac237 36436009

[4] Australian and New Zealand College of Anaesthetists. A framework for perioperative care in Australia and New Zealand. 2021. https://www.anzca.edu.au/safety-advocacy/standards-of-practice/the-perioperative-care-framework. Accessed 31 October 2022.

[5] Thillainadesan J, Hilmer S, Close J, Kearney L, Naganathan V (2019). Geriatric medicine services for older surgical patients in acute hospitals: results from a binational survey. Australas J Ageing.

[6] Joughin AL, Partridge JSL, O’Halloran T, Dhesi JK (2019). Where are we now in perioperative medicine? Results from a repeated UK survey of geriatric medicine delivered services for older people. Age Ageing.

[7] Sbai M, Martin F, Partridge J, Dhesi J (2020). Comprehensive geriatric assessment (CGA) in the perioperative setting: the current state of play. J R Coll Physicians Edinb.

[8] Partridge JS, Harari D, Martin FC (2017). Randomized clinical trial of comprehensive geriatric assessment and optimization in vascular surgery. BJS.

[9] Shipway D, Koizia L, Winterkorn N, Fertleman M, Ziprin P, Moorthy K (2018). Embedded geriatric surgical liaison is associated with reduced inpatient length of stay in older patients admitted for gastrointestinal surgery. Future Healthc J.

[10] Thillainadesan J, Aitken SJ, Monaro SR (2022). Geriatric comanagement of older vascular surgery inpatients reduces Hospital-Acquired geriatric syndromes. JAMDA.

[11] Braude P, Goodman A, Elias T (2017). Evaluation and establishment of a ward-based geriatric liaison service for older urological surgical patients: proactive care of older people undergoing surgery (POPS)-Urology. BJU Int.

[12] Thu K, Nguyen HP, Gogulan T (2021). Care of older people in surgery for general surgery: a single centre experience. ANZ J Surg.

[13] Ibitoye SE, Braude P, Carter B (2023). Geriatric assessment is associated with reduced mortality at 1-year for older adults admitted to a major trauma centre: a prospective observational study. Ann Surg.

[14] Vilches-Moraga A, Fox J (2018). Geriatricians and the older emergency general surgical patient: proactive assessment and patient centred interventions. Salford-POP-GS. Aging Clin Exp Res.

[15] Partridge JS, Healey A, Modarai B, Harari D, Martin FC, Dhesi JK (2021). Preoperative comprehensive geriatric assessment and optimisation prior to elective arterial vascular surgery: a health economic analysis. Age Ageing.

[16] Loh M. Perioperative Care of Older People. 2022. https://anzsgm.org/wp-content/uploads/2022/05/ANZSGM-Position-Statement-Perioperative-Care-of-Older-People_-FINAL.pdf. Accessed 8 February 2023.

[17] Mohanty S, Rosenthal RA, Russell MM, Neuman MD, Ko CY, Esnaola NF (2016). Optimal Perioperative Management of the geriatric patient: a best practices Guideline from the American College of Surgeons NSQIP and the American Geriatrics Society. J Am Coll Surg.

[18] Skivington K, Matthews L, Simpson SA (2021). A new framework for developing and evaluating complex interventions: update of Medical Research Council guidance. BMJ.

[19] Horton T, Illingworth J, Warburton W. The spread challenge. How to support the successful uptake of innovations and improvements in health care. Health Foundation. 2018. https://www.health.org.uk/publications/the-spread-challenge. Accessed 1 March 2023.

[20] Jasper EV, Dhesi JK, Partridge JS, Sevdalis N (2019). Scaling up perioperative medicine for older people undergoing surgery (POPS) services; use of a logic model approach. Clin Med (Lond).

[21] May CR, Johnson M, Finch T (2016). Implementation, context and complexity. Implement Sci.

[22] Thillainadesan J, Hilmer SN, Fleury AM, Naganathan V (2022). New horizons in the perioperative care of older adults. Age Ageing.

[23] Dhesi J, Moonesinghe SR, Partridge J (2019). Comprehensive Geriatric Assessment in the perioperative setting; where next?. Age Ageing.

[24] de Las Casas R, Meilak C, Whittle A (2021). Establishing a perioperative medicine for older people undergoing surgery service for general surgical patients at a district general hospital. Clin Med (Lond).

[25] Waring J, Martin GP, Hartley P, Partridge JS, Dhesi JK (2023). Implementing a perioperative care of older people undergoing surgery (POPS) service: findings from a multi-site qualitative implementation study. Age Ageing.

[26] Yin RK (2003). Case study research: design and methods.

[27] Schofield JW. Increasing the generalizability of qualitative research. In: Gomm R, Hammersley M, Foster P, editors. Case study method. SAGE; 2000.

[28] Tong A, Sainsbury P, Craig J (2007). Consolidated criteria for reporting qualitative research (COREQ): a 32-item checklist for interviews and focus groups. Int J Qual Health Care.

[29] Damschroder LJ, Aron DC, Keith RE, Kirsh SR, Alexander JA, Lowery JC (2009). Fostering implementation of health services research findings into practice: a consolidated framework for advancing implementation science. Implement Sci.

[30] Carter JE, Pyati S, Kanach FA (2018). Implementation of perioperative music using the consolidated framework for implementation research. Anesth Analg.

[31] Kakar E, van Ruler O, van Straten B (2022). Implementation of music in colorectal perioperative standard care—barriers and facilitators among patients and healthcare professionals. Colorectal Dis.

[32] Crain N, Qiu C-Y, Moy S (2021). Implementation science for the adductor canal block: a new and adaptable methodology process. World J Orthop.

[33] Deftereos I, Hitch D, Butzkueven S (2022). Implementing a standardised perioperative nutrition care pathway in upper gastrointestinal cancer surgery: a mixed-methods analysis of implementation using the Consolidated Framework for implementation research. BMC Health Serv Res.

[34] Lane-Fall MB, Beidas RS, Pascual JL (2014). Handoffs and transitions in critical care (HATRICC): protocol for a mixed methods study of operating room to intensive care unit handoffs. BMC Surg.

[35] Lambert-Kerzner AC, Aasen DM, Overbey DM (2019). Use of the consolidated framework for implementation research to guide dissemination and implementation of new technologies in surgery. J Thorac Dis.

[36] The NHS Constitution for England. https://www.gov.uk/government/publications/the-nhs-constitution-for-england/the-nhs-constitution-for-england. Accessed 20 September 2022.

[37] Olmos-Vega FM, Stalmeijer RE, Varpio L, Kahlke R (2023). A practical guide to reflexivity in qualitative research: AMEE Guide 149. Med Teach.

[38] Barry CA, Britten N, Barber N, Bradley C, Stevenson F (1999). Using reflexivity to optimize teamwork in qualitative research. Qual Health Res.

[39] Greenhalgh T, Robert G, Macfarlane F, Bate P, Kyriakidou O (2004). Diffusion of innovations in service organizations: systematic review and recommendations. Milbank Q.

[40] Kitson A, Harvey G, McCormack B (1998). Enabling the implementation of evidence based practice: a conceptual framework. BMJ Qual Saf.

[41] Plsek PE, Wilson T (2001). Complexity, leadership, and management in healthcare organisations. BMJ.

[42] Waltz TJ, Powell BJ, Fernández ME, Abadie B, Damschroder LJ (2019). Choosing implementation strategies to address contextual barriers: diversity in recommendations and future directions. Implement Sci.

[43] Hull L, Goulding L, Khadjesari Z (2019). Designing high-quality implementation research: development, application, feasibility and preliminary evaluation of the implementation science research development (ImpRes) tool and guide. Implement Sci.

[44] Hunter SC, Kim B, Kitson AL (2023). Mobilising implementation of i-PARIHS (Mi-PARIHS): development of a facilitation planning tool to accompany the Integrated Promoting Action on Research Implementation in Health Services framework. Implement Sci Commun.

[45] Damschroder LJ, Reardon CM, Widerquist MAO, Lowery J (2022). The updated Consolidated Framework for Implementation Research based on user feedback. Implement Sci.

