# Supplementary material for: The implementation of a perioperative medicine for older people undergoing surgery service: a qualitative case study
Source: BMC Health Serv Res. 2024 Mar 15;24:345. doi: 10.1186/s12913-024-10844-0 (PMC10943911; doi:10.1186/s12913-024-10844-0)
Supplement: Supplementary file 1 — Supplementary Material 1 [file 12913_2024_10844_MOESM1_ESM.docx]

**Supplementary Information**

Additional file 1: Consolidated criteria for reporting qualitative research (COREQ) checklist

Additional file 2: Disciplines represented and interview response rates

Additional file 3: Interview schedule – initial version

Additional file 4: Interview schedule – revised version

Additional file 5: Research team roles and experience

Additional file 6: Additional illustrative quotations

Additional file 7: CFIR diagram

**Additional file 1**

Consolidated criteria for reporting qualitative research (COREQ) checklist

| **No. Item** | **Guide questions / description** | **Section reported in / further details** |
| --- | --- | --- |
| **Domain 1: Research team and reﬂexivity** | | |
| Personal characteristics | | |
| 1. Interviewer/facilitator | Which author/s conducted the interview or focus group? | *Case selection, sampling and recruitment;*  *Data collection* |
| 2. Credentials | What were the researcher’s credentials? E.g., PhD, MD | *Case selection, sampling and recruitment;*  *Additional file 5* |
| 3. Occupation | What was their occupation at the time of the study? | *Case selection, sampling and recruitment;*  *Additional file 5* |
| 4. Gender | Was the researcher male or female? | Not reported. |
| 5. Experience and training | What experience or training did the researcher have? | *Case selection, sampling and recruitment;*  *Data collection;*  *Additional file 5* |
| Relationship with participants | | |
| 6. Relationship established | Was a relationship established prior to study commencement? | *Case selection, sampling and recruitment* |
| 7. Participant knowledge of the interviewer | What did the participants know about the researcher? e.g., personal goals, reasons for doing the research | *Case selection, sampling and recruitment* |
| 8. Interviewer characteristics | What characteristics were reported about the interviewer/facilitator? e.g., Bias, assumptions, reasons and interests in the research topic | *Additional file 5* |
| **Domain 2: study design** | | |
| Theoretical framework | | |
| 9. Methodological orientation and theory | What methodological orientation was stated to underpin the study? e.g., grounded theory, discourse analysis, ethnography, phenomenology, content analysis | *Study design;*  *Theoretical framework* |
| Participant selection | | |
| 10. Sampling | How were participants selected? e.g., purposive, convenience, consecutive, snowball | *Case selection, sampling and recruitment* |
| 11. Method of approach | How were participants approached? e.g., face-to-face, telephone, mail, email | *Case selection, sampling and recruitment* |
| 12. Sample size | How many participants were in the study? | *Participant characteristics* |
| 13. Non-participation | How many people refused to participate or dropped out? Reasons? | *Participant characteristics*;  Reasons for non-participation not collected or reported. |
| Setting | | |
| 14. Setting of data collection | Where was the data collected? e.g., home, clinic, workplace | Not reported.  Participants chose time and place for interview to be conducted via the Zoom platform (home or work). |
| 15. Presence of non-participants | Was anyone else present besides the participants and researchers? | Not reported.  Non-participants may have been present in shared office spaces but were not actively involved in observing interviews. |
| 16. Description of sample | What are the important characteristics of the sample? e.g., demographic data, date | *Participant characteristics;*  *Additional file 2* |
| Data collection | | |
| 17. Interview guide | Were questions, prompts, guides provided by the authors? Was it pilot tested? | *Data collection;*  *Additional file 3;*  *Additional file 4* |
| 18. Repeat interviews | Were repeat interviews carried out? If yes, how many? | Not reported.  Not carried out. |
| 19. Audio/visual recording | Did the research use audio or visual recording to collect the data? | *Data collection* |
| 20. Field notes | Were ﬁeld notes made during and/or after the interview or focus group? | *Data collection* |
| 21. Duration | What was the duration of the interviews or focus group? | *Participant characteristics* |
| 22. Data saturation | Was data saturation discussed? | Not reported. |
| 23. Transcripts returned | Were transcripts returned to participants for comment and/or correction? | Not returned. |
| **Domain 3: analysis and ﬁndings** | | |
| Data analysis | | |
| 24. Number of data coders | How many data coders coded the data? | *Data analysis* |
| 25. Description of the coding tree | Did authors provide a description of the coding tree? | Not provided. |
| 26. Derivation of themes | Were themes identiﬁed in advance or derived from the data? | *Data analysis* |
| 27. Software | What software, if applicable, was used to manage the data? | *Data collection;*  *Data analysis* |
| 28. Participant checking | Did participants provide feedback on the ﬁndings? | *Discussion* |
| Reporting | | |
| 29. Quotations presented | Were participant quotations presented to illustrate the themes/ﬁndings? Was each quotation identiﬁed? e.g., participant number | *Factors relevant to implementation* |
| 30. Data and ﬁndings consistent | Was there consistency between the data presented and the ﬁndings? | *Factors relevant to implementation* |
| 31. Clarity of major themes | Were major themes clearly presented in the ﬁndings? | *Factors relevant to implementation* |
| 32. Clarity of minor themes | Is there a description of diverse cases or discussion of minor themes? | *Factors relevant to implementation* |

**Additional file 2**

Disciplines represented and interview response rates

|  | **Case 1** | **Case 2** | **Case 3** |
| --- | --- | --- | --- |
| **Participants (n, %)** | 23 (41) | 17 (30) | 16 (29) |
| **Response rate (%)** | 51 | 27 | 67 |
| **Area of specialty (n, %)** | | | |
| Geriatrician | 2 (9) | 3 (18) | 1 (6) |
| Surgeon | 2 (9) | 7 (41) | 3 (19) |
| Anaesthetist | 3 (13) | 0 (0) | 2 (13) |
| Geriatric medicine trainee | 2 (9) | 3 (18) | 0 (0)* |
| Surgical trainee | 2 (9) | 1 (6) | 1 (6) |
| Advanced nursing practitioner | 1 (4) | 0 (0)* | 0 (0)* |
| Clinical nurse specialist | 1 (4) | 0 (0)* | 1 (6) |
| Surgical matron | 1 (4) | 0 (0) | 0 (0) |
| Surgical ward nurse | 2 (9) | 1 (6) | 1 (6) |
| Occupational therapist | 1 (4) | 0 (0)* | 2 (13) |
| Physiotherapist | 1 (4) | 1 (6) | 1 (6) |
| Pharmacist | 1 (4) | 0 (0)* | 1 (6) |
| General practitioner | 1 (4) | 0 (0)* | 0 (0)* |
| Clinical response team nurse practitioner | 1 (4) | 0 (0)* | 1 (6) |
| Board / Executive | 1 (4) | 1 (6) | 1 (6) |
| General Manager | 1 (4) | 0 (0)* | 1 (6) |

*specialty/discipline not part of the POPS service for this case

POPS, perioperative medicine for older people undergoing surgery.

**Additional file 3**

Interview schedule – initial version

*General questions (pertaining to each of the contextual factors)*

How important was this factor in the successful initial implementation of POPS?

- Prompts: was, and how, was it identified as such?; who was involved in recognising its importance?; what barriers did this factor bring up?; how were barriers overcome?; how did this factor support establishment of POPS?

How important has this factor been for ongoing implementation of POPS and the growth of the service?

- Prompts: how successful would growth have been without it?; how has the role of this factor changed with time?; who are the people involved in making this factor ‘work’?; where has this factor hindered development?; how has hinderance been overcome?; how has this factor been embraced/harnessed for ongoing growth?

*Focused questions (relevant to specific contextual factors)*

‘Buy-in’ from board, surgeons, anaesthetists and other key stakeholders

‘Buy-in’ from ward teams

Buy-in from all stakeholders

What does the term, ‘buy-in’, mean to you?

How and by whom was the level and degree of ‘buy-in’ assessed during initial implementation of POPS?

How was ‘buy-in’ supported or encouraged in the early days of POPS?

- Prompts: who, what, how, when

What was the level and degree of ‘buy-in’ when POPS was in its early days?

How do you see people involved in POPS ‘buy-in’ to the service?

How has this changed over time?

How is ‘buy-in’ to POPS supported or encouraged nowadays?

- Prompts: active vs passive process; who does it?; who displays it?

How else could ‘buy-in’ be encouraged?

Who are the people who most needed to ‘buy-in’ to make POPS successful?

Who are the people who least needed to ‘buy-in’ to make POPS successful?

What do you think was/is the ‘magic wand’ to achieve the ‘buy-in’ required for POPS’ success?

Skills and engagement of medical and AHP staff equipped to deliver CGA and optimisation in the perioperative period

(Engagement – see above re buy-in)

What specific skills are required of medical and AHP staff to deliver CGA and optimisation in the perioperative period?

How were the necessary skills to deliver CGA and optimisation identified, prior to implementing POPS?

How were the disciplines/people with these skills identified, prior to implementing POPS?

- How were these disciplines/people recruited?

How have the necessary skills changed over time?

- Prompts: have there been new/unexpected skills felt required to deliver CGA and optimisation?; have there been skills that were thought necessary and subsequently felt to not be?; were there skills initially necessary that are now less-so?

What disciplines are a necessity to successfully implement POPS?

What disciplines are a necessity to successfully sustain POPS?

IT infrastructure, technical support and physical space

Support from informatics

How does physical infrastructure allow POPS’ success?

What physical infrastructure is necessary?

What physical infrastructure is optional?

How does IT infrastructure and informatics allow POPS’ success?

How did POPS develop the necessary infrastructure (physical, IT, informatics) to support initial implementation?

- Prompts: who was involved in this?; how was this identified?; how was this funded?; did you have to create new IT/paper-based systems or pathways?

How has the surrounding infrastructure changed with time and how has POPS had to adjust as a result of this?

How does infrastructure support or hinder the day-to-day service provision of the POPS team?

How does infrastructure and informatics support data collection relevant to POPS?

How does data affect day-to-day care?

Funding

How was POPS funded when it was first implemented?

- Prompts: how was the initial budget devised?; how was funding sourced?; who was involved in sourcing funding?; what were some of the difficulties in obtaining funding?; what made obtaining funding easier?; how vital was the initial funding in establishing the service?

How has the POPS funding model changed with time?

What has contributed to changes to the POPS funding model?

Professional jurisdictions, norms and codes of behaviour

How did professional jurisdictions, norms and codes of behaviour support initial implementation of POPS?

How were these supportive jurisdictions, norms and codes demonstrated?

How were potential enablers identified?

What strategies were used to maximise this support?

How did professional jurisdictions, norms and codes of behaviour hinder initial implementation of POPS?

How were these hindering jurisdictions, norms and codes demonstrated?

How were potential barriers identified?

What strategies were used to overcome these barriers?

How do professional jurisdictions, norms and codes of behaviour support ongoing implementation of POPS?

How are these supportive jurisdictions, norms and codes demonstrated?

How are potential enablers identified?

What strategies are used to maximise this support?

How do professional jurisdictions, norms and codes of behaviour hinder ongoing implementation of POPS?

How are these hindering jurisdictions, norms and codes demonstrated?

How are potential barriers identified?

What strategies are used to overcome these barriers?

Readiness for change within the clinical team and the organisation (open to new ways of delivering collaborative patient-centred care)

How was the readiness for change within the clinical team assessed, when planning for initial implementation of POPS?

- Prompts: who assessed this?; how did they do so?; were any tools used to assess readiness for change?; how was this information fed back to the people driving change?

How was the readiness for change within the organisation assessed, when planning for initial implementation of POPS?

- Prompts: who assessed this?; how did they do so?; were any tools used to assess readiness for change?; how was this information fed back to the people driving change?

How were barriers to change within the clinical team identified during the initial stages of implementation of POPS?

How were barriers to change within the clinical team overcome in initial implementation of POPS?

- Prompts: who was involved in this?; how did they go about dealing with the barriers?; what resources were involved to assist with this?; were any of the barriers insurmountable?; if barriers were insurmountable, how was this managed?

How were barriers to change within the organisation identified during the initial stages of implementation of POPS?

How were barriers to change within the organisation overcome in initial implementation of POPS?

- Prompts: who was involved in this?; how did they go about dealing with the barriers?; what resources were involved to assist with this?

How were insurmountable barriers within the organisation managed?

As POPS evolves during ongoing implementation, how are ‘new’ barriers to change within the clinical team identified? How are these managed?

How were enablers of change within the clinical team identified and taken advantage of during the initial implementation of POPS?

- Prompts: who was involved in this?; how did they go about utilising the enablers?; what resources were involved to assist with this?

As POPS evolves during ongoing implementation, how are ‘new’ enablers of change within the clinical team identified? How are these utilised?

As POPS evolves during ongoing implementation, how are ‘new’ enablers of change within the organisation identified? How are these utilised?

Promotion of collaborative working (on individual, team, and organisational level)

Avoidance of silo working and poor communication (whether due to IT or other systems)

How was collaborative working and communication promoted during initial implementation of POPS?

How important to initial implementation of POPS was collaborative working and communication?

- Prompts: why?; what did collaboration/communication enable?; how would POPS have evolved if collaboration/communication was different?; who did the collaborating and communicating?

How were any barriers to collaboration/communication identified during initial implementation of POPS?

How were these barriers overcome?

How were any enablers of collaboration/communication identified during initial implementation of POPS?

How were these enablers harnessed?

How does collaborative working/communication for POPS ‘play out’, in day-to-day practice these days?

- Prompts: what works well?; what is difficult?; who is involved in supporting communication/collaboration?; are there any ‘must have’ factors that support communication/collaboration?; how do rotating clinicians impact on communication/collaboration?; what resources help communication/collaboration?; what resources hinder communication/collaboration?

Trusted informal and formal peer review and accountability with willingness to engage with feedback process

How was development of peer review and feedback processes considered during initial implementation of POPS?

How did peer review and feedback processes impact upon initial implementation of POPS?

How does peer review and feedback occur in the current day-to-day practice of POPS?

How important has peer review and feedback been to the evolution of POPS?

Scheduled protected teaching sessions

How was the role and creation of scheduled protected teaching sessions considered during initial implementation of POPS?

- Prompts: who was involved in conceptualising?; who was involved in determining content?; how did consideration of teaching sessions help/hinder POPS’ implementation?; were teaching sessions felt to be a necessity or a ‘luxury’?; who funded the time for students and teachers?; which disciplines were targeted for protected teaching time?

How did having scheduled protected teaching sessions contribute to the successful initial implementation of POPS?

How are schedule protected teaching sessions integrated as part of the day-to-day running of the current POPS service?

Access to expertise in quality improvement

What quality improvement approaches were utilised in the initial implementation of POPS?

How were quality improvement approaches utilised in the initial implementation of POPS?

Who provided quality improvement expertise and how were these people identified?

How were quality improvement resources funded during initial implementation of POPS?

How does quality improvement expertise inform day-to-day running of POPS?

How does quality improvement expertise inform ongoing implementation and future development of POPS?

**Additional file 4**

Interview schedule – revised version

Opening questions:

What is your role at [hospital name] and how do you work [as part of / alongside] the POPS team?

How long have you been working [as part of / alongside] the POPS team?

Additional prompt questions:

What are your reflections on the way the POPS service has developed with time?

What do you think are the key things at [hospital name] that have enabled the POPS service to develop?

Are there any factors that have acted as barriers to the POPS service developing?

What do you see as the key, critical success factors of POPS?

Is there any advice you’d give another health service planning to set up a POPS service?

**Additional file 5**

Research team roles and experience

| **Name** | **Role** | **Experience relevant to this qualitative case study** |
| --- | --- | --- |
| Margot Lodge | Conceived study  Designed and wrote study protocol  Designed and modified interview schedule  Recruited participants  Conducted interviews  Analysed and interpreted data  Generated results and tables  Wrote and revised manuscript | Consultant geriatrician working in perioperative care of older people  PhD student |
| Jugdeep Dhesi | Revised study protocol  Reviewed interview schedule  Identified potential study participants  Assisted with participant recruitment  Study participant  Reviewed and interpreted results and tables  Revised manuscript | Clinical Lead – POPS service at Case 1  Consultant geriatrician working in perioperative care of older people  Pioneer of original POPS service |
| David Shipway | Identified potential study participants  Assisted with participant recruitment  Study participant  Reviewed and interpreted results and tables  Revised manuscript | Clinical Lead – POPS service at Case 2  Consultant geriatrician working in perioperative care of older people |
| Philip Braude | Identified potential study participants  Assisted with participant recruitment  Study participant  Reviewed and interpreted results and tables  Revised manuscript | Clinical Lead – POPS service at Case 2  Consultant geriatrician working in perioperative care of older people |
| Catherine Meilak | Identified potential study participants  Assisted with participant recruitment  Study participant  Reviewed and interpreted results and tables  Revised manuscript | Clinical Lead – POPS service at Case 3  Consultant geriatrician working in perioperative care of older people |
| Judith Partridge | Identified potential study participants  Assisted with participant recruitment  Reviewed and interpreted results and tables  Revised manuscript | Clinical Lead – POPS service at Case 1  Consultant geriatrician working in perioperative care of older people |
| Nadine Andrew | Reviewed and interpreted results and tables  Revised manuscript | Implementation science expertise |
| Velandai Srikanth | Reviewed and interpreted results and tables  Revised manuscript | Research expertise |
| Darshini Ayton | Conceived study  Identified and guided methodology  Revised study protocol  Reviewed interview schedule  Supervised first three interviews  Supervised data analysis and interpretation  Generated results and tables  Revised manuscript | Implementation science expertise  Qualitative methods expertise |
| Chris Moran | Conceived study  Revised study protocol  Reviewed interview schedule  Analysed and interpreted data  Generated results and tables  Revised manuscript | Consultant geriatrician working in acute care of the elderly and orthogeriatric care  Research expertise |

**Additional file 6**

Additional illustrative quotations

|  | **Case 1** | **Case 2** | **Case 3** |
| --- | --- | --- | --- |
| **Intervention characteristics** | | | |
| *Evidence strength and quality*  Clinician and manager perceptions of the evidence supporting the POPS service. Includes ‘external sources’ of evidence (e.g., peer-reviewed literature) and ‘internal sources’ (e.g., local patient outcomes data from the participants’ health services). | Theme: Belief in the evidence for the POPS service supports implementation, with external evidence especially important at sites wanting to newly implement POPS. | | |
|  | “If we're being purist about it, no, we don't have multi-site RCT data. But have we got a strong enough evidence base to support this being the appropriate way forward with buy-in? Yes, we do … [As a] surgeon said, we're never going back to prove that parachutes work, but we're not going to stop using them.”  (POPS clinical lead) | “I think everybody thinks it is quite rich in terms of its investment … the benefit around length of stay and the evidence base [that] sits behind it is very, very powerful … So [changing practice] really wasn’t that difficult.”  (Executive) | “I had seen the data, I’d seen the research and I’d seen it in practice … it made sense to me that you needed to take a multidisciplinary approach to yield an acceptable outcome for these patients.”  (Executive) |
| *Adaptability*  The degree that the POPS service can be tailored so that it can meet each case’s local needs and capabilities. | Theme: The POPS service can be adapted to enable its implementation to be tailored to local needs and resources. | | |
|  | “[The POPS service has] had to evolve how it approaches or manages the patients. It used to be [only] older patients they’d be involved with [and] they’re now increasingly involved with younger patients with complex frailty.”  (Occupational therapist) | “I don’t believe it’s efficient to make one size fit all for every clinical scenario. I don’t think the intervention needs to be the same.”  (POPS clinical lead) | “I still wasn't getting [referred] all the patients I wanted so bit by bit I have changed what I've been doing and … integrating myself into [the surgical unit’s] day-to-day work very subtly.”  (POPS clinical lead) |
| **Outer setting** | | | |
| *Patient needs and resources*  The level of understanding within the health service of the needs of older people undergoing surgery, and the barriers and facilitators to meeting those needs. | Theme: Understanding and prioritising the needs and priorities of older people undergoing surgery supports the implementation of POPS services. | | |
|  | “We have daily contact with the POPS team and we found it really useful because ... all our patients are getting more complex and the more you know about their surgeries, the more you can have an input and realise how many more [patients] have so many other medical issues.”  (Pharmacist) | “Older people need more holistic care and not just, ‘oh we’ll fix the fracture and send them on their way’ … it is quite hard to look after older people and if we all work together we do a better job … [POPS results in] this team mentality and everyone is enthusiastic.”  (POPS fellow) | “The nurses particularly like having POPS because [previously if] they’d had [a patient] they’ve been worried about … they might have said ‘oh I’m a bit worried’ … [to] the surgeon but now they’ll often come and find us … I think for them it’s a relief that there’s a team that will particularly go and look at those more medical concerns.”  (POPS clinical nurse specialist) |
| *Cosmopolitanism (external networking)*  Networking of the health service with other organisations. | Theme: At health services without a POPS service, networking with external well-established POPS services and their clinical leads facilitates implementation. | | |
|  | “The other thing that is useful is that the [POPS] fellowship … equips people with the competencies to be able to [deliver POPS] … elsewhere and that's what we've seen here … a lot of the services in the UK, Bristol, Oxford have been previous fellows at Guy’s and St Thomas'.”  (Geriatrician) | “[The POPS clinical leads] came here from London and they were both pretty well known in their fields at the time and came with clear aims to set up this service and with perioperative medicine as a priority.”  (Geriatrician) | “I don’t think I had a lot of formal training but I’ve learnt as I’ve gone, along with the support from [case 1 POPS clinical lead] who has done it before.”  (POPS clinical lead) |
| *External policies and incentives*  The role of external initiatives to spread interventions. | Theme: Financial incentives can assist with the implementation of the POPS service but are not an essential factor. | | |
|  |  | “[To receive the Best Practice Tariff, patients are] supposed to have a frailty score within 72 hours [of admission] so we tagged on that they should get a full geriatric assessment.”  (Physiotherapist) |  |
| **Inner setting** | | | |
| *Networks and communications*  The nature and quality of relationships and connections between individuals, clinical units and teams that interact with the POPS service. | Theme: Networks at multiple organisational levels facilitate sharing of vision and define team members’ roles, which enables delivery of POPS services. | | |
|  | “Two-way learning worked well [to build the model of care] … [for example] if we ask a question about medical stuff, [the POPS clinical lead] will explain it … And equally if there was stuff that [the POPS clinical lead] didn't understand about the discharge pathways or surgery pathways [we would give that education].”  (Physiotherapist) | “Having easy lines of communication between our team and [the POPS] team has been quite useful because more often than not they’ll see the patient once [only] … whereas we’ll be seeing that patient continually so … if there’s anything else that crops up we can then speak to them again.”  (Physiotherapist) | “[The POPS clinical lead and I] communicate very effectively and I know historically [at other health services] there’s been fragmentation between the way anaesthetists do their preoperative assessment and the way the POPS team do and we were quite careful to work together to avoid that becoming a problem and have achieved that completely.”  (Anaesthetist) |
| Implementation climate | | | |
| *Tension for change*  The extent to which clinicians and managers feel the care provided to older people undergoing surgery needs to change. | Theme: The presence of an unmet clinical need results in a tension for change that facilitates the implementation of POPS services. | | |
|  | “We tend not to have that much physician back-up on this site … I think that’s made a huge difference because we don’t have the physicians to rely on and to ask so therefore there was that niche area that POPS picked up on and it’s been amazingly helpful.”  (Anaesthetist) | “They plugged the gap … [it] was incredibly difficult to get any alternative input into patients, especially when you’re [wanting] to do an operation today or tomorrow … [because] our other speciality colleagues were not as engaged and not as convenient … so when someone offered to provide this service for us, we said fine, it’s going to fill a big need so let’s just do it. We’re all very happy with what’s happened.”  (Surgeon) | “[POPS] works on what is essentially an elective site and a site that doesn’t have a ‘hot’ A&E so, therefore they don’t have acute medicine and the full remit of acute services that can pick up these complications. So there is a gap in the market that [POPS] has conveniently filled.”  (Anaesthetist) |
| *Compatibility*  The level of alignment between the POPS service and a clinician or manager’s goals, skill mix and values. The perceived risks and benefits of introducing the POPS service. | Theme: POPS services may be perceived as a threat when clinicians do not see a clinical need the service can meet; POPS services are thus viewed as a risk to autonomy or territory. | | |
|  | “I would look at POPS in two aspects. One is the pre-assessment and the outpatient preop assessment … and that is the bit where I always find it slightly bizarre that you’ve got a physician seeing a patient who’s having an anaesthetic, and they’re giving anaesthetic advice.”  (Anaesthetist) | “The other thing that has been problematic is the relationship between the anaesthetic team and [POPS]. Some of the anaesthetists think, ‘what's the point [of POPS assessment]?’ … I think that that can be a barrier to spreading [POPS] because people can use some of this as empire building.”  (Surgeon) | “We thought, ‘okay here is a physician’ … people viewed it with scepticism saying, ‘what can she tell us that we don’t know already?’ But people have realised the value of the service and I think patients have benefited enormously, the service has benefited enormously.”  (Surgeon) |
| *Learning climate*  A climate with time and space for leaders to feel and express fallibility, team members to feel valued and able to assist leaders, and which safely enables trial and error. | Theme: POPS clinical leads role-model and drive a learning climate that supports implementation. | | |
|  | “[POPS] feels much more supportive than other ward rounds, [it] is much more integrational [sic]. People feel that they’re able to speak up and ask questions so I think that’s what it brings to [improved safety and quality].”  (Surgical matron) | “The junior doctors on the wards have loved [POPS commencing] because anything that’s slightly complex with an older patient they can just ask a senior person what the answer is.”  (Physiotherapist) | “I’ve got that vascular surgical knowledge that [the POPS clinical lead] doesn’t have as much of so that’s worked quite well. [She may ask] what [I] think, or I say [I don’t think that’s] a good idea [and] that’s worked well because she’s got the medical knowledge and I’ve got a bit more of the vascular knowledge.”  (POPS clinical nurse specialist) |
| Readiness for implementation | | | |
| *Available resources*  Financial, the capacity to educate stakeholders, physical and time-based resources that are dedicated for implementation and use of the POPS service. | Theme: The implementation of POPS services can be enabled by adequate resources for staffing, financial support and education. | | |
|  | “[Our POPS service is] fortunate … teaching hospitals with good reputations attract good people and so they're well resourced.”  (Board member) | “We’ve tried to always maintain that flexibility and that adaptability [in our staffing] and I think that’s one of the advantages … we have enough staff that we can provide flexibility, time off, study leave and cover all the areas needed but you have to have a critical mass of staff, you can’t be one single practitioner trying to deliver an entire service.”  (POPS clinical lead) | “[The POPS clinical lead] is a one-man team and works like five people … I think [health service] management … just dismiss that point … she works so hard because she has to … but when she’s meant to finish at 4.45 or 5 o’clock and she’s still there at 8 o’clock … management don’t realise that we’re really short staffed because we’re getting the job done but we’re getting the jobs done in our own time.”  (Surgical trainee) |
| *Access to knowledge and information*  The ability for users of the POPS service to be easily educated about what the POPS service is and how to engage with it. | Theme: Knowledge and information about the why, what and how of POPS services is necessary for implementation and can be provided through multiple channels according to local needs and capabilities. | | |
|  | “Education’s important. I went to a meeting and [the POPS clinical lead] was talking [about POPS] and it was amazing. I'd never heard anything about it before … That’s very important, when you're just starting out, to make yourself known widely.”  (Anaesthetist) | “[The POPS clinical lead] approached us, he showed some evidence and we were all aware of the evidence that was emerging about the [length of] stay reduction … and he came [and said] this is my experience, this is what I can do for you, please send me your patients.”  (Surgeon) | “We were quite confused when POPS turned up as to what they were here for and what they were doing. It took quite a while of the POPS consultant explaining her role as to what she was here for and what she was doing and what we could come to her with.”  (Physiotherapist) |
| **Characteristics of individuals** | | | |
| *Knowledge and beliefs about the intervention*  Individual clinicians’ understanding of the rationale for the POPS service. The skills and enthusiasm that individual clinicians have in referring to and engaging with the POPS service. | Theme: Understanding the rationale for the POPS service drives initial adoption, and positive experience with the POPS service supports enthusiasm for ongoing implementation. | | |
|  | “If it’s something that’s a good idea and everybody thinks ‘oh my God that’s amazing’ … then they do support you and I think that’s what happened with the POPS team, they saw it early on and thought this is a fantastic idea because there’s a need for it, surgeons like it, it was good for patients and that’s why it worked so well.”  (Anaesthetist) | “[One surgical department] recognised they had a terrible length of stay and so contacted [the POPS clinical lead] and said we’ve heard about your service from other departments, would you come and do some sessions for us.”  (POPS fellow) | “We probably kept on asking [the POPS clinical lead] about any patient we could possibly ask her about on the ward because she was there a lot and it was just nice to have someone to ask, so she had to … educate us a bit that her role didn’t include every single patient on the ward from a medical perspective … I think it’s been a learning curve but we’ve learnt what her role is and now we’re finding it incredibly useful.”  (Physiotherapist) |
| **Process** | | | |
| *Engaging*  The process of attracting and involving individual clinicians and managers to implement and use the POPS service. | Theme: Engagement of individuals to implement and utilise POPS services is supported by a flexible and proactive approach to involving early adopters from a range of disciplines who see the benefits of the service. | | |
|  | “Several of my vascular [anaesthesia] colleagues … did POPS clinics and worked with [the now POPS clinical lead] when they were all trainees … training together builds that link from the start and you know who these people are and there's a … relationship already in place.”  (Anaesthetist) | “There's obviously a bit of charm offensive going in. But ultimately the proof's in the pudding, and you just have to go and [provide the perioperative care] … [And] a new patient with a new surgeon is a great opportunity to get another customer … [there is a] cycle of … charming surgeons [and] achieving … the hearts and minds war, with [the surgeons] realising that they get value … You've got to be committed to it and it's not for the faint hearted.”  (POPS clinical lead) | “[When I started I was] finding out … what was currently going on, where my colleagues thought I could help, where I thought I could help … the first few weeks was very much about getting to know who I was working with and finding out what was important to them, [and being] wary that you don't just come in with ideas without understanding what's currently going on.”  (POPS clinical lead) |
| *Champions*  Individuals who believe in the POPS service, actively associate themselves with the POPS service and who are dedicated to what is required to implement the POPS service. | Theme: POPS clinical leads are essential champions of the implementation of the POPS service. | | |
|  | “[The POPS clinical leads] put a lot of time and effort and support into their team but, most significantly … not just the medical team but everybody else. As nurses we matter. They don’t stop pushing, encouraging … So you feel that they value your work and your input just as much as they would a [geriatric medicine] registrar’s … POPS is like a family as well as a workplace … I’ve never had a bunch of consultants like them. It is quite rare I think, especially if you’ve worked with surgeons, you just can’t believe that consultants can be like this. They definitely are a big driving force in the success of how the team works.”  (POPS clinical nurse specialist) | “[The] two consultants that run the service are quite young, ambitious, quite forward-thinking and I think that really helps, they’re quite ambitious to get geriatrics embedded in trauma and … into the [non-trauma] wards as well. I think their drive, having those very proactive consultants has helped.”  (Physiotherapist) | “Some weeks [the POPS clinical lead] will work 12½ hour days all week just to try and keep our head above water … she will always … see the patients, she will always find the time to teach the juniors and support them … they’ve all got her mobile number and it’s like any questions or any worries don’t hesitate get in touch with me … so … it’s just pure hard work and determination, love for the job and for the patients … she’s an amazing leader … [and] doctor … if I ever had 1% of her knowledge then I think I’d be quite happy.”  (POPS clinical nurse specialist) |

RCT, randomised controlled trial; POPS, perioperative medicine for older people undergoing surgery; A&E, Accident and Emergency

**Additional file 7**

CFIR diagram

**
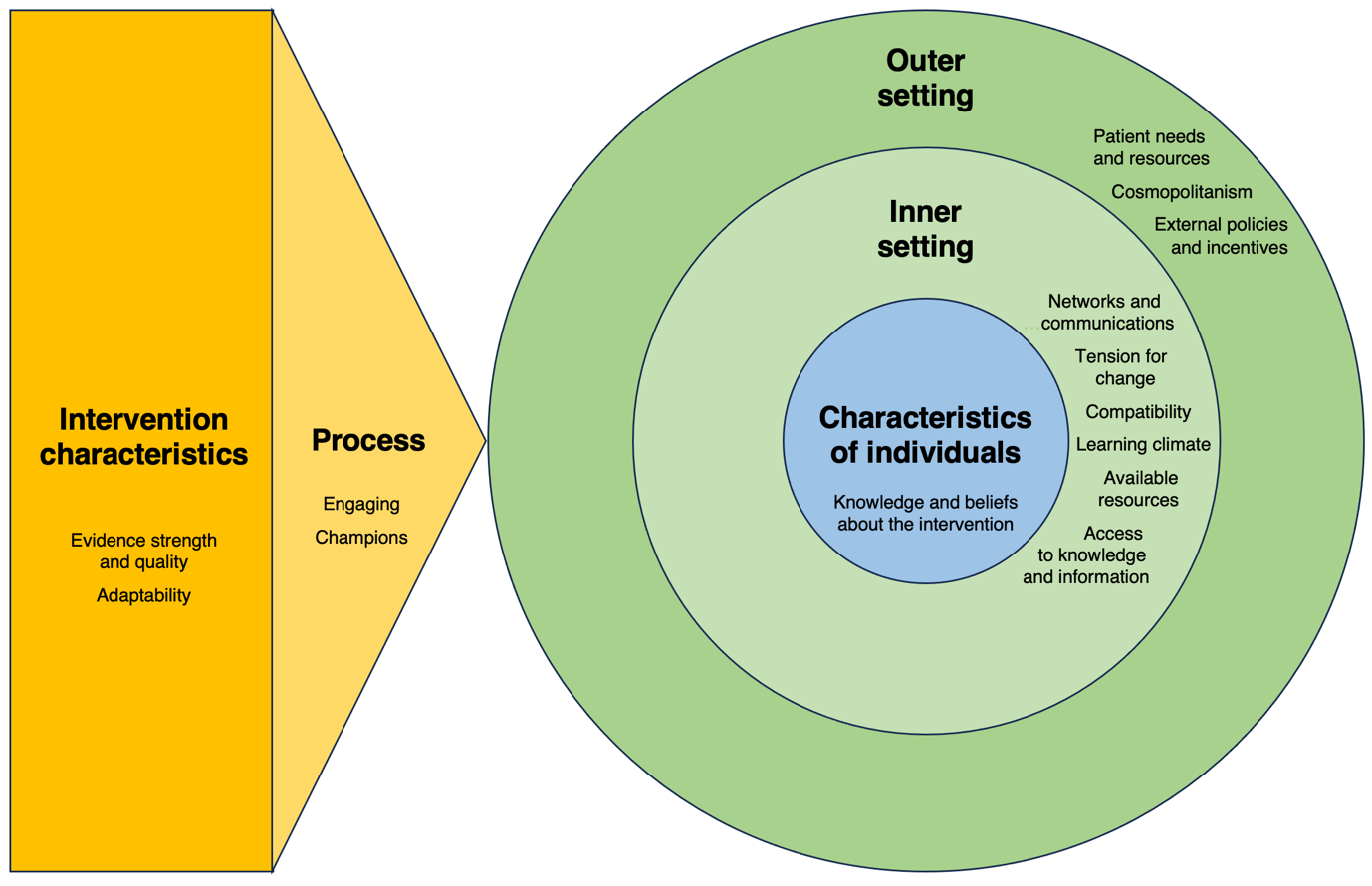
**

Figure 1: CFIR constructs relevant to the implementation of a POPS service.
